# Effects of fasting on endothelial function in type 2 diabetes mellitus patients: cohort study

**DOI:** 10.3389/fcvm.2025.1646786

**Published:** 2026-01-07

**Authors:** Feten Lamti, Salma Messous, Marwa Toumia, Imen Trabelsi, Randa Dhaoui, Khaoula Bel Haj Ali, Ekram Hajji, Bilal Benamor, Adel Sekma, Rahma Jaballah, Hajer Yaakoubi, Asma Zorgati, Kaouthar Beltaief, Houda Ben Soltane, Zied Mezgar, Mariem Khrouf, Imen Ben Abdallah, Amira Sghaier, Nahla Jerbi, Rabie Razgallah, Ilhem Hallara, Wahid Bouida, Mohamed Habib Grissa, Jamel Saad, Hamdi Boubaker, Riadh Boukef, Fadoua Neffati, Nabil Sakly, Zohra Dridi, Mohamed Amine Msolli, Asma Sriha, Ines Khochtali, Semir Nouira

**Affiliations:** 1 Research Laboratory LR12SP18, Monastir University, Monastir, Tunisia; 2 Emergency Department, Haj Ali Soua Regional Hospital, Monastir, Tunisia; 3 Emergency Department, Fattouma Bourguiba University Hospital, Monastir, Tunisia; 4 Department of Endocrinology and Internal Medicine, Fattouma Bourguiba University Hospital, Monastir, Tunisia; 5 Emergency Department, Sahloul University Hospital, Sousse, Tunisia; 6 Emergency Department, Farhat Hached University Hospital, Sousse, Tunisia; 7 Laboratory of Biochemistry and Toxxicology, Fattouma Bourguiba University Hospital, Monastir, Tunisia; 8 Emergency Department, Taher Sfar University Hospital, Mahdia, Tunisia; 9 DACIMAConsulting, Tunis, Tunisia; 10 Department of Imaging and Interventional Radiology, Fattouma Bourguiba University Hospital, Monastir, Tunisia; 11 Departement of Immunology, Fattouma Bourguiba University Hospital, Monastir, Tunisia; 12 Departement of Cardiology, Fattouma Bourguiba University Hospital, Monastir, Tunisia; 13 Departement of Epidemiology and Preventive Medicine, Fattouma Bourguiba University Hospital, Monastir, Tunisia

**Keywords:** diabet mellitus type 2, endothelial function, fasting (Ramadan), homocysteine, reactive hyperaemia index

## Abstract

**Background:**

The aim of this study was to investigate the effect of Ramadan fasting (RF) on markers of endothelial function and metabolic parameters in patients with type 2 diabetes mellitus (T2DM) and healthy controls.

**Methods:**

This cohort study involved 305 subjects: 167 T2DM and 138 healthy volunteers subjects. The following parameters were evaluated: body mass index (BMI), diet inflammatory index (DII), serum glucose and lipid profile and high-sensitivity C-reactive protein (hs-CRP) during three separate visits, before Ramadan (baseline), the last week of Ramadan, and during the last week of the month following Ramadan. In each visit, reactive hyperaemia index (RHI) in all participants was assessed. In diabetic patients, serum homocyteine (Hcys) was mesured at baseline and during RF. The primary endpoint was change in RHI, a validated and non-invasive measure of endothelial function. The secondary end point was change in clinical, metabolic parameters, and Hcys. Analyses used non-parametric tests and multivariable regression with multiple imputation.

**Results:**

RF significantly decreased RHI [−0.2 UI (95% CI −0.3 to −0.09); *p* < 0.001] and increased serum homocysteine [1.2 µmol/L (95% CI 0.7–1.8); *p* < 0.001] in T2DM compared to baseline levels.RF did not significantly change RHI in healthy volunteers. A significant increase in blood glucose (*p* < 0.001) and LDL cholesterol (*p* = 0.003) was observed during Ramadan compared to baseline, whereas HDL cholesterol, DII, and BMI decreased. Among healthy volunteers, similar changes in metabolic parameters were observed, with hs-CRP increasing during Ramadan compared to baseline (*p* = 0.01).

**Conclusion:**

RF seems to be associated with unfavorable effects on endothelial function in diabetic patients but not in healthy subjects. RHI testing could help identifying diabetic patients who should not observe RF or should be closely monitored during fasting.

## Introduction

During Ramadan, millions of Muslims with cardiovascular risk factors abstain from food, water, beverages, oral medicine, and smoking from sunrise to sunset (1). Ramadan intermittent fasting involves specific dietary and lifestyle modifications, including altered meal timing, food quantity and quality, nocturnal eating, reduced meal frequency, sleep disruption, and reduced physical activity (2, 3). It has been shown that Ramadan fasting (RF) could be associated with metabolic changes in patients with diabetes (4–6); while many studies report minimal variations on glycemic control (7–12), a recent meta-analysis revealed that RF has the potential to improve HbA1c and fasting blood glucose in people with diabetes mellitus type 2 T2DM (13).

Beyond its glycemic effects, RF may also help prevent endothelial dysfunction—a key contributor to long-term vascular complications in diabetes, by reducing oxidative stress, and systemic inflammation, enhancing nitric oxide bioavailability, improving lipid and glucose metabolism, and promoting favorable hormonal and circadian rhythm adaptations. Few studies have evaluated endothelial outcomes in diabetic and non-diabetic populations (14, 15). Flow-mediated dilation (FMD) and reactive hyperemia-peripheral arterial tonometry (RH-PAT) are both an established method to assess non-invasively endothelial function. Unlike FMD, RH-PAT is operator-independent and easy to use compared with FMD (16), enabling efficient large-scale assessment. In this study, we evaluated the effects of RF on endothelial function using RH-PAT in T2DM patients and healthy adults.

## Patients and methods

### Design

This prospective cohort study included adult healthy subjects and patients with T2DM. They were recruited from university and non-university medical centers. Volunteers' participants were recruited, using convenience sampling, at the hospital using bulletin boards advertisements seeking research project healthy volunteers. Diabetic patients were screened in outpatient clinics (endocrinology, internal medicine, family medicine) when they presented for scheduled follow-up. Selection was based on the participant's decision to fast. Exclusion criteria included age under 18 years, diabetics who remain unstable despite antidiabetic treatment, current or previous (14 days) use antidepressants, inability to give informed consent, or terminal chronic disease. After screening, the study design and requirements were thoroughly explained to the participants. Ethical approval for this study was granted by the Research Ethics Committee of Faculty of Medicine of Monastir, under protocol number IORG 0009738 N°114/OMB0990-0279. All procedures performed in studies involving human participants were in accordance with the ethical standards of the institutional and/or national research committee and with the Helsinki Declaration and its later amendments or comparable ethical standards. This trial was registered at ClinicalTrial.gov (NCT05331443).

### Intervention

The study lasted four years (2019–2022) with three separate assessment visits in each year: 1) the week before Ramadan which represented the baseline period (before-R); 2) the last week of Ramadan (during-R); 3) and during the last week of the month following Ramadan (after-R). The duration of fasting was approximately 15 h from sunrise to sunset (the time of abstinence from food) during a 30-d period. The assessment in each of the three visits involved physical exam and blood sampling for standard biological tests including, glycemia, lipid profile, and high-sensitivity C-reactive protein (hs-CRP). Serum homocysteine determination using liquid chromatography electrospray tandem mass spectrometric method was performed before-R and during-R. Body weight and height were performed, and body mass index (BMI) was calculated as body weight (kg) divided by squared height in meters (m^2^). Physical examination included systolic and diastolic blood pressure, and heart rate, Additionally, participants completed a questionnaire on their diet. No special nutritional regimen was applied to the participants during the study. All subjects were encouraged to continue their usual lifestyle and activities. All significant clinical events requiring physician consultation or hospital admission during the study period were reported. For patients with comorbidity, compliance to current treatment was assessed by the attending physician based on interview and pill count. Venous blood samples were collected from the enrolled participants during the three time points. The time of blood sampling in the study was 9–10 am, at which all participants were fast. And for the purpose of the study, patients were asked to take their last meal (“sehour”) between 11 o'clock pm and midnight. At the two protocol visits, nutritional data was obtained using three days food record questionnaire that was confirmed by certified dietitian. We assessed the inflammatory potential of the diet using the dietary inflammatory index (DII) developed by Shivappa et al. (17). The DII score ranges from low values (minimum ≈ −8) that represent an anti-inflammatory diet to high values (maximum ≈ +8) that indicate a proinflammatory diet. This index was created based on an extensive literature review in order to assess the effect of food parameters (foods/nutrients) on the inflammatory markers (18).

### Assessment of endothelial function by non-invasive peripheral arterial tonometry

Peripheral endothelial function was assessed by RH-PAT using the RH-PAT method (Health Tronics, Tunisia) performed after blood sampling at each protocol visit. Room temperature was maintained at all time during the study between 21 °C and 24 °C; restrictive clothing that could interfere with blood flow to the arms, watches or rings or other jewelry on the hands and fingers were removed and the patient was comfortably seated or allowed to lie down in the study room for at least 15 min to reach a relaxed cardiovascular steady-state. A blood pressure cuff was placed on one upper arm (study arm), while the other arm served as a control (control arm); PAT probes were placed on one finger of each hand for continuous recording of the PAT signal. After a 10-min equilibration period, the blood pressure cuff was inflated to supra systolic pressures (60 mm Hg upper systolic blood pressure and, in all case, *N* < 200 mm Hg) for 5 min. Then the cuff was deflated, while PAT recording continued for 10 min. PAT data were analyzed by a computer in an operator-independent manner as previously described. As a measure of reactive hyperemia, a PAT reactive hyperaemic index (RHI) was calculated as the ratio of the average amplitude of the PAT signal over a 1-min time interval starting 1-min after cuff deflation divided by the average amplitude of the PAT signal of a 3.5-min time period before cuff inflation (baseline). Subsequently, RHI value from the study arm was normalized to the control arm. Endothelial dysfunction was defined as a RHI ≤1.6.

### Primary and secondary outcomes

The primary endpoint was the change in RHI, a validated and non-invasive measure of endothelial function, during-R compared to before-R period. Secondary endpoint is the change of serum homocysteine and metabolic parameters including glycemia, serum lipid profile, BMI, DII, and hs-CRP.

### Statistical analysis

A pre-study sample-size calculation indicated that a sample of 500 patients was sufficient to detect a 10% improvement in reactive hyperaemia index, with a power of 80% and *α* = 0.05.

Continuous variables were tested for normal distribution by Kolmogorov–Smirnov test. The quantitative variables were expressed by their means with the estimation of their confidence intervals at 95%. A non-parametric test (Friedman test: Dunn's Test with Bonferonni adjustment) was used to compare non Gaussian quantitative variables before, during and after Ramadan. Two-sided *p* values of less than 0·05 were considered to indicate statistical significance. We included odds ratio (ORs) and 95% confidence intervals (CIs) in the comparison of during-R primary and secondary outcomes. A multivariable logistic regression was used to investigate the association between RHI changes and prespecified variables including age, gender, comorbidities, baseline and Ramadan induced change of BMI, DII, glycemia, serum lipid profile, hs-CRP, and serum homocysteine. We used chained equations to impute missing values in the predictor variables with multiple imputation. Because of the small sample size, only variables with a univariate *P* value of less than 0.20 were entered in to this analysis. A backward elimination algorithm was then applied with the use of a *P* value of less than 0.05. All analyses were done using the Statistical Package for Social Sciences software (SPSS version 20.0).

## Results

In total, 305 participants were included, 167 patients in diabetic T2DM group, and 138 healthy volunteers (volunteers group).

### Healthy volunteers

**Figure 1:**
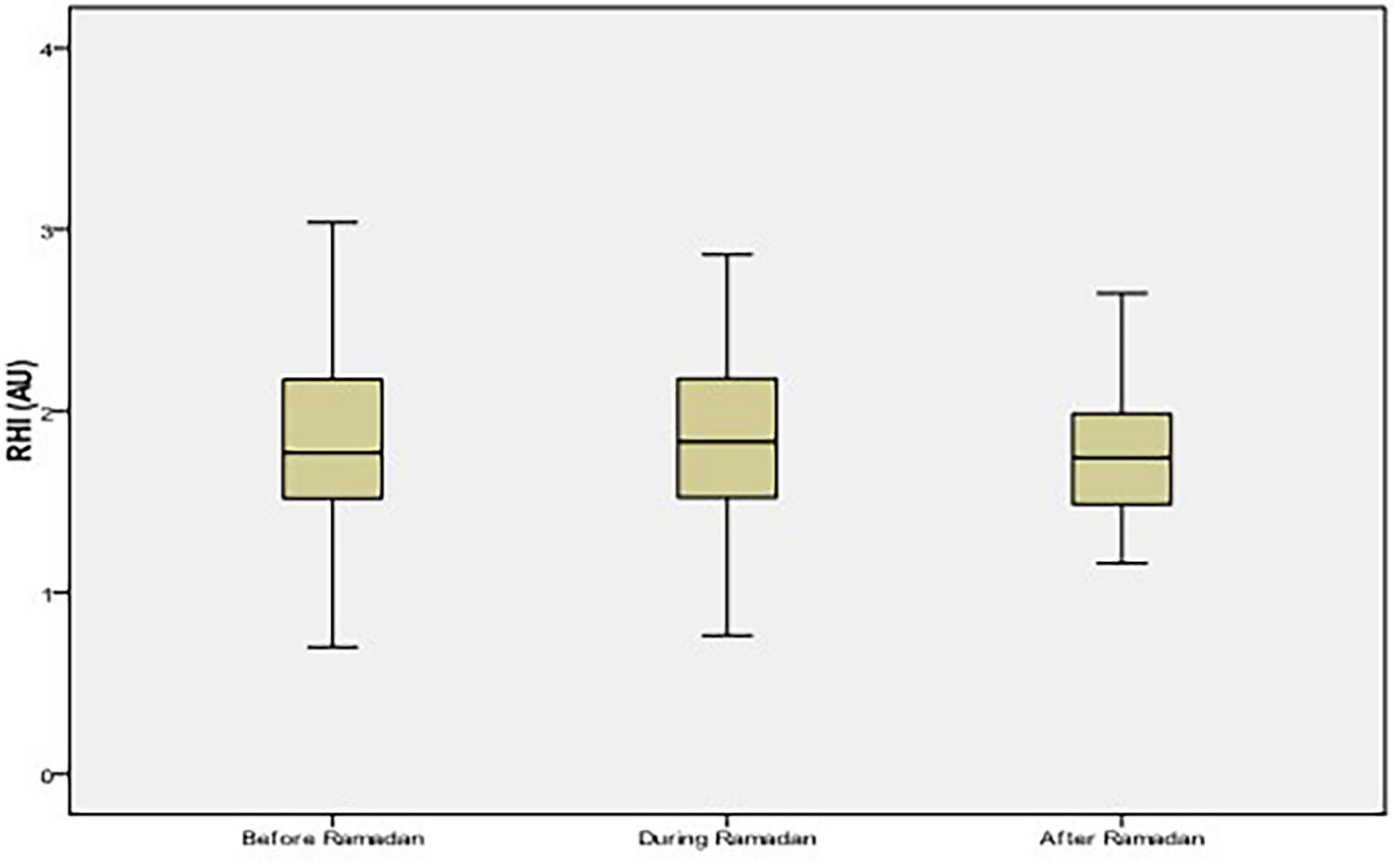
Reactive Hyperemia Index (RHI) values among healthy volunteers. No significant difference of RHI values between the three periods (*p* = 0.08). AU, arbitrary unit.

The mean age was 47 ± 13 years (21–81 years) with a male/female ratio 0.3. Apart from a history of smoking found in 4% of subjects, no other comorbidities were reported. An RHI ≤ 1.6, indicating impaired endothelial function, was present in 45% of the group. The changes of RHI during-R and after -R were not significant compared to before-R (Figure 1). Changes in metabolic parameters during-R and after-R compared to before-R period are shown in Table 1. No significant changes were observed with regard to triglycerides and HDL cholesterol, while the serum glucose, total cholesterol, and LDL cholesterol increased significantly during Ramadan compared to before-R. BMI and DII decreased significantly during-R compared to before-R (*p* < 0.001). The hs-CRP levels increased during-R compared to before-R and decreased after-R (*p* = 0.01).

**Table 1:** Changes of clinical and biological parameters during the three study periods (before, during, and after Ramadan) among healthy volunteers (*n* = 138).

Clinical and biological parameters	Before-Ramadan	During-Ramadan	After-Ramadan	*p*
Mean [95% CI]				
Systolic blood pressure (mmHg)	121 [117–125]	117 [113–120]*	119 [115–124]	0.01
Diastolic blood pressure (mmHg)	76 [74–78]	76 [74–79]	77 [74–80]	0.53
Heart rate **(**bpm**)**	70 [68–73]	72 [70–74]	71 [69–74]	0.11
BMI, (kg/m^2^)	29.8 [28.8–31.0]	29.6 [28.5–30.7]*	29.7 [28.7–30.8]	<0.001
Hs-CRP	1.9 [1.5–2.43]	2.7 [1.5–4.5]	1.9[1.5–2.5]	0.01
DII	−4.1 [(−4.6)-(−3.7)]	−4.35 [(−4.7)-(−3.9)]	−3.29 [(−3.5)-(−3)]^$^^,^^£^	<0.001
Glycemia, (mmol/L)	5.6 [5.3–5.9]	6.4 [6–7]*	5.9 [5.2–6.8]^$^	<0.001
Cholesterol, (mmol/L)	4.6 [4.5–4.8]	4.9 [4.6–5.2]*	4.6 [4.3–4.9] ^$^	0.02
Triglycerides, (mmol/L)	1.3 [1.1–1.4]	1.4 [1.2–1.6]	1.3 [1.1–1.5]	0.53
HDL-c, (mmol/L)	1.2 [1.1–1.3]	1.1 [1.08–1.2]	1.2 [1.1–1.2]	0.83
LDL-c, (mmol/L)	3.2 [3–3.4]	3.4 [3.2–3.7]*	3.2 [2.9–3.4] ^$^	0.01

BMI, body mass index; Hs-CRP, high-sensitivity C-reactive protein; DII, dietary inflammatory index; HDL-c, high-density lipoprotein cholesterol; LDL-c, low-density lipoprotein cholesterol.

* *p* < 0.05 during Ramadan vs. before Ramadan,

$ *p* < 0.05 during Ramadan vs. after Ramadan,

£ *p* < 0.05 before Ramadan vs. after Ramadan.

### T2DM patients

**Figure 2:**
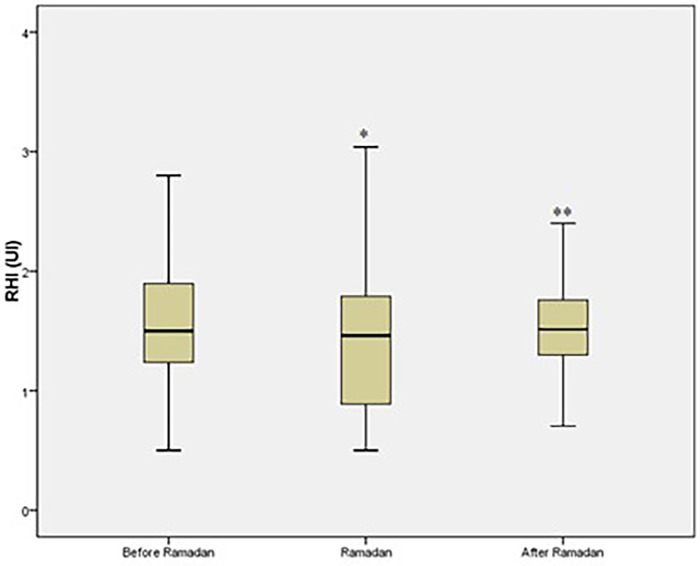
Reactive Hyperemia Index (RHI) values among T2DM patients. *p ≤ 0.05 vs. before Ramadan. **p ≤ 0.05 vs. during Ramadan. AU: arbitrary unit.

The mean age was 59 ± 9 years with a male/female ratio 0.62. Most diabetic patients were overweight (15.1%) or obese (84.9%). History of hypertension was present in 124 patients (74.3%), history of coronary artery disease in 17 patients (10%), dyslipidemia in 81 patients (48.5%), and smoking in 14 patients (8.4%). Endothelial dysfunction was observed in 76% of the group.Compared to levels before-R, RHI significantly decreased during-R [difference −0.2 (95% CI −0.3 to −0.09); *p* < 0.001] and then significantly increased after-R [difference 0.11 (95% CI 0.005–0.22); *p* < 0.001] (Figure 2).

**Figure 3:**
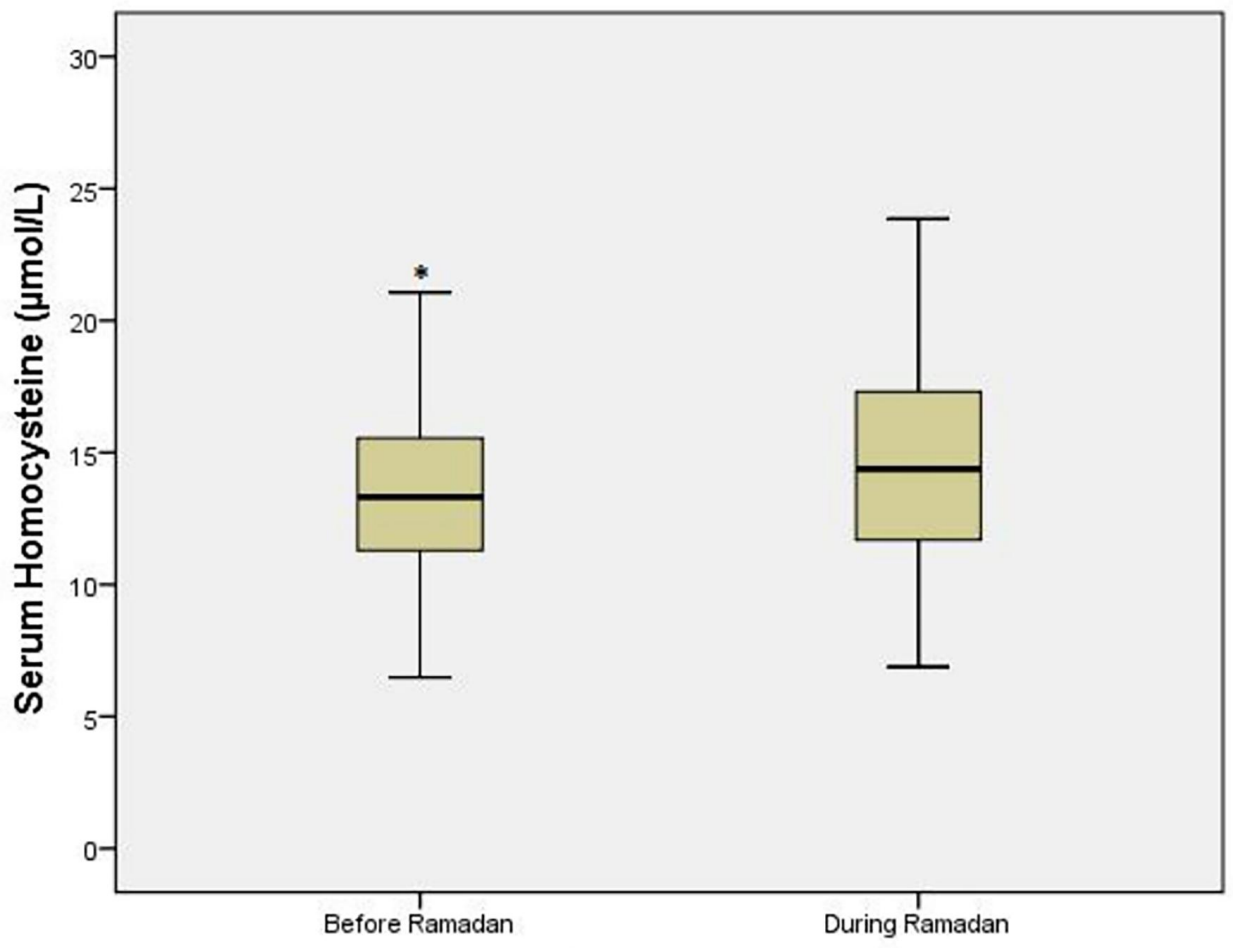
Homocysteine values in T2DM patient. *p ≤ 0.05 vs. during Ramadan.

Homocysteine increased significantly during-R [difference 1.2 mmol/L (95% CI 0.7–1.8); *p* < 0.001] (Figure 3). Multivariate analysis did not identify any variable independently associated with RHI changes, except delta homocysteine (homocysteine before-R minus homocysteine during-R), which was associated with a fasting RHI decrease [OR (95% CI) 2.7 (0.98–7.69), *p* = 0.054]. Changes in metabolic parameters are shown in Table 2.

**Table 2:** Changes of biological parameters during the three study periods (before, during, and after ramadan) in the diabetic patients (*n* = 167).

Clinical and biological parameters	Before-Ramadan	During-Ramadan	After-Ramadan	*p*
Mean [95% CI]				
Systolic blood pressure (mmHg)	140 [130–160]	139 [127–150]	136 [124–150]^$^^,^^£^	0.001
Diastolic blood pressure (mmHg)	80 [70–87]	80 [73–86]	80 [73–84]	0.32
Heart rate (beats)	77 [71–85]	76 [69–83]	76 [68–80]	0.11
BMI, (kg/m^2^)	31.4 [30.4–32.5]	31.2 [30.1–32.3]	30.9 [29.9–32.1]^$^^,^^£^	<0.001
Hs-CRP,	4.7 [2.3–8.7]	3 [2.1–4.2]	3.1 [2–4.7]	0.4
DII	−4.4 [(−4.8)-(−3.9)]	−5 [(−5.5)- (−4.6)]*	−4 [(−4.5)- (−3.5)]^$^	<0.001
Glycemia, (mmol/L)	8.9 [7.9–9.9]	10.3 [9.1–11.5]*	8.9 [7.9–10]^$^	<0.001
Cholesterol, (mmol/L)	4.5 [4.2–4.8]	4.6 [4.3–4.9]	4.2 [4 -4.6]^$^	0.001
Triglycerides, (mmol/L)	1.7 [1.5–1.9]	1.77 [1.5–2]	1.63 [1.4- 1.8]	0.71
HDLc, (mmol/L)	1 [0.9–1.1]	0.9 [0.9–1]*	1 [0.9 -1.7]^$^	0.001
LDLc, (mmol/L)	2.9 [2.6–3.1]	3.1 [2.8–3.4]*	3.2 [2.9–3.5]^$^	0.003

BMI, body mass index; Hs-CRP, high-sensitivity C-reactive protein; DII, dietary inflammatory index; HDL-c, high-density lipoprotein cholesterol; LDL-c, low-density lipoprotein cholesterol.

* *p* < 0.05 during Ramadan vs. before Ramadan,

$ *p* < 0.05 during Ramadan vs. after Ramadan,

£ *p* < 0.05 before Ramadan vs. after Ramadan.

During Ramadan, we observed a significant increase in blood glucose levels (difference 1.4 mmol/L (95% CI 0.4–1.8; *p* < 0.001) and LDL cholesterol [difference 0.16 mmol/L (95% CI 0.01–0.31); *p* = 0.003]. We also observed a significant decrease during Ramadan of HDL cholesterol [difference −0.1 mmol/L (95% CI −0.15 to −0.005); *p* = 0.001], DII [–0.61 (95% CI −0.96 to −0.08); *p* < 0.001], and BMI [–0.4 kg (95% CI −0.96 to −0.08); *p* < 0.001]. Total cholesterol, triglycerides and hs-CRP did not significantly change. As in healthy volunteers, systolic blood pressure decreased significantly during-R compared to before-R. After Ramadan, a return to baseline values was observed for most of these parameters (Table 2). The percentage of hypoglycemic events among diabetic patients was low and similar during the three study periods. No diabetic patient was admitted to the Emergency Department for hyperglycemia exacerbation during the study period.

## Discussion

In diabetic patients, RF was associated with a significant decrease in RHI and alteration of metabolic parameters, including glycemia and lipid profile, alongside reductions in BMI and DII. Most variables returned to pre-Ramadan levels after RF. Notably, no interaction was observed between the decrease in RHI and changes of metabolic parameters, BMI, DII, or hs-CRP. In healthy volunteers, RF did not significantly affect RHI but was associated with increases in glycemia, cholesterol, and LDL-c levels.

It is widely recognized that diabetes is one of the most frequent disorders worldwide, with rising incidence over recent decades (19). T2DM is an independent risk factor for cardiovascular disease and accelerates changes in vascular structure and function, especially endothelial function (20). Interventions improving diabetes may thus also benefit endothelial function. Increasing evidence supports RF's positive effects on glycemic control, though data on endothelial function remain limited. Caloric restriction and intermittent fasting have been shown to improve cardiovascular outcomes by decreasing insulin resistance and increasing insulin sensitivity, potentially enhancing endothelial function, especially in obese patients (21). In healthy subjects, Bastani et al. and Esmaeilzadeh et al. showed respectively a significant decrease in oxidative stress markers (22) and increase of acethylcholine induced hyperemic skin reactions (23). In diabetic patients, data are limited to one retrospective cohort study performed on 26 T2DM patients using intercellular adhesion molecule-1 (ICAM-1) as a biological marker of endothelial function (24). Other studies in patients with cardiovascular risks or obesity have reported improvements in nitric oxide levels, triglycerides, HDL cholesterol, and FMD after RF (14, 25, 26). In contrast, our study observed increased homocysteine levels and decreased RHI in diabetic patients (27), independent of baseline characteristics or metabolic changes suggesting other factors may influence endothelial function during RF (28). Short-term glucose fluctuations during RF can trigger proinflammatory pathways and oxidative stress (29–31), and transient hyperglycemia has been shown to induce even more vascular damage than sustained hyperglycemia (32). Thus, the decrease in RHI during fasting may be explained by these glucose fluctuations, which promote oxidative stress and endothelial injury, and by potential alterations in autonomic function and vascular tone independent of other metabolic changes.

This study has several limitations that deserve mention. First, The sample size was smaller than initially calculated, due to limited eligible participants, recruitment challenges, and logistical constraints, however, a *post-hoc* power analysis based on the observed effect size and variability of RHI demonstrated that the achieved sample size still provided adequate statistical power (≥80%) to detect significant differences in the primary endpoint. Extrapolation of our results to type I diabetic patients should be specifically investigated. Second, there has been no agreement on the ideal test for the evaluation of endothelial dysfunction (33). Measurements of circulating endothelial biomarkers is often used in clinical trials; they are currently recommended with regard to their accuracy and easy interpretation by clinicians (34, 35). In the present study we used peripheralarterial tonometry and serum homocysteine to assess endothelial function and we found consistent results. Use of additional biomarkers of antioxidant capacity and oxidative stress in our study would strengthen the validity of our results, however, their cost was a limiting factor. In addition, previous studies largely demonstrated a significant positive correlation between these measurements methods and both were correlated with cardiovascular events (36, 37).

In conclusion, our findings suggest that Ramadan fasting, a form of intermittent fasting has unfavorable effects on endothelial function in diabetic patients but not in healthy subjects. This effect was independent of baseline characteristics or metabolic derangements occurring during RF. Our findings suggest to consider RHI testing for better identifying in diabetic patients who should not observe RF and provide individualized guidance regarding fasting safety, potential risks, and strategies to minimize vascular complications, such as careful glycemic monitoring, lifestyle adjustments, and close follow-up during Ramadan.

## Data Availability

The raw data supporting the conclusions of this article will be made available by the authors, without undue reservation.
